# When stress exceeds developmental capacity: rethinking cell death in bronchopulmonary dysplasia

**DOI:** 10.3389/fped.2026.1809960

**Published:** 2026-03-13

**Authors:** Manuela Marega, Cho-Ming Chao

**Affiliations:** 1 Centre for Clinical and Translational Research (CCTR), Helios University Hospital Wuppertal, Witten/Herdecke University, Wuppertal, Germany; 2 Cardio-Pulmonary Institute (CPI), Universities of Giessen and Marburg Lung Center (UGMLC), Member of the German Center for Lung Research (DZL), Justus Liebig University Giessen, Giessen, Germany; 3 St. Louise Women’s and Children’s Hospital, St. Vincenz-Kliniken, Paderborn, Germany

**Keywords:** apoptosis, bronchopulmonary dysplasia, developmental stress buffering, lung development, mitochondrial dysfunction, non-apoptotic cell death, pulmonary hypertension

## Introduction

Bronchopulmonary dysplasia (BPD) is a chronic lung disease of premature infants, characterized by impaired alveolar and vascular development. One of its most frequent and severe comorbidities is pulmonary hypertension, which is associated with a markedly increased risk of mortality (1, 2). Over the past decade, increasing evidence has implicated multiple forms of programmed cell death, including apoptosis, pyroptosis, necroptosis, ferroptosis, and autophagy-related cell death. Here, autophagy is primarily a stress-buffering, pro-survival developmental process whose dysregulation can indirectly contribute to cell death in BPD (3–5). Recent comprehensive reviews have detailed the involvement of multiple programmed cell death pathways in BPD. However, these processes are often considered parallel injury mechanisms occurring alongside established pathological features, rather than indicators of a deeper disruption of lung developmental programs (5, 6).

Notably, non-apoptotic forms of programmed cell death such as pyroptosis, necroptosis, and ferroptosis are not part of physiological lung organogenesis. Normal embryonic and fetal lung development relies predominantly on tightly regulated, non-inflammatory apoptosis and autophagy to allow precise tissue remodeling while preserving a sterile environment (7). The emergence of lytic and highly inflammatory death pathways in the immature lung does not reflect misused developmental mechanisms, but rather the failure of developmental systems to contain stress within physiological apoptotic boundaries (7). Thus, the emergence of inflammatory and lytic death pathways in the developing lung represents a qualitative shift in cell fate control, rather than an exaggeration of physiological remodeling. This distinction is often underemphasized in current models of BPD.

While recent work has framed mitochondria as central regulators coordinating multiple cell death pathways in BPD (5), this perspective largely interprets pathway activation as an integrated response to injury. In contrast, we propose that the emergence of non-apoptotic, lytic forms of cell death reflects a failure of developmental stress buffering in the immature lung, whereby apoptotic resolution becomes insufficient due to metabolic and mitochondrial immaturity. This distinction—between regulated pathway coordination and loss of apoptotic control due to developmental state—has not been explicitly addressed in mitochondria-centric syntheses.

From this model, we predict that interventions restoring apoptotic competence during early alveolar stages should prevent engagement of inflammatory lytic death pathways. This hypothesis is directly testable using precision-cut lung slice (PCLS) models across defined developmental stages.

## Developmental buffering and failure of apoptotic control

In this Opinion, we propose a developmental timing—and state-dependent model in which cell death becomes pathological at the moment immature lung cells lose the capacity to resolve stress through regulated apoptosis. Lung development follows a tightly orchestrated temporal program that depends on adequate metabolic support, mitochondrial function, and controlled inflammatory signaling (8, 9). When this buffering capacity is exceeded, cells are forced into pathological death modes that should not be engaged during development. Here, “timing” refers not only to chronological age, but to the developmental and metabolic state of lung cells at the moment of environmental challenge. In the immature lung, apoptotic failure likely reflects a convergence of metabolic and clearance constraints: limited ATP availability and redox imbalance can blunt caspase activation, while an overabundance of stressed or dying cells may exceed local phagocytic capacity, promoting secondary necrosis and spillover into lytic pathways (5, 10–12).

Importantly, the trajectory leading to BPD does not begin at birth. Maternal infection and intrauterine inflammation are strongly associated with increased BPD risk, suggesting that fetal lung cells may enter postnatal life with altered inflammatory, metabolic, and mitochondrial set points (13). Prenatal inflammatory exposure can prime immune signaling and mitochondrial stress responses without causing overt structural damage (14). Extracellular vesicles and exosomes have emerged as plausible mediators of this priming, transmitting inflammatory and metabolic cues across the maternal–fetal interface and shaping how neonatal lung cells respond to subsequent insults (15).

## Prenatal priming and postnatal stress as trajectory disruptors

Following preterm birth, life-saving interventions such as hyperoxia and mechanical ventilation impose non-physiological conditions on a lung that is still growing and differentiating. These interventions cause oxidative stress, mechanical tension, and inflammatory signaling that converge on mitochondrial dysfunction at a time when angiogenesis, epithelial differentiation, and immune maturation are still ongoing (5). In this context, apoptotic pathways may become insufficient or dysfunctional due to metabolic constraints, redox imbalance, or impaired caspase activity. As a result, cells may default to non-apoptotic, lytic forms of death that amplify inflammation and tissue injury.

## Consequences of developmental failure

From this perspective, the coexistence of multiple cell death modalities in BPD does not represent coordinated activation of alternative programs; rather, it is a signature of developmental failure. In line with this, autophagy is part of the developmental buffering machinery; when overwhelmed or dysregulated, it can cease to be protective and facilitate irreversible cell death. Pyroptosis, necroptosis, and ferroptosis emerge as pathological escape routes when the immature lung loses the ability to maintain stress responses within developmental limits. This framework helps explain why different lung compartments—epithelial, endothelial, immune, and mesenchymal—exhibit divergent death and survival responses to similar insults, leading to loss of coordination across the developing lung. This model generates testable predictions, including that interventions enhancing mitochondrial resilience or apoptotic competence during early postnatal windows may prevent transition to pathological death modes.

Pulmonary hypertension can be framed within the same framework as the vascular manifestation of this disrupted developmental process. Endothelial injury, mitochondrial dysfunction, and premature endothelial cell death impair angiogenesis and reduce vascular surface area, increasing pulmonary vascular resistance (5). The vascular compartment is therefore not a downstream victim of parenchymal injury, but an integral participant in the same disrupted developmental trajectory. BPD-associated pulmonary hypertension further reflects the propagation of developmental failure across the vascular compartment, reinforced by hypoxia, inflammation, and structural arrest.

## Discussion and concluding remarks

Viewed through this lens, cell death in BPD is not simply excessive, but mismanaged in time and constrained by immature cellular state. Therapeutic strategies aimed at indiscriminately blocking individual death pathways are unlikely to succeed. Interventions that preserve mitochondrial function, enhance developmental buffering capacity, and maintain stress responses within physiological apoptotic boundaries may offer a more effective means of preventing both parenchymal and vascular sequelae of BPD, for example by supporting mitochondrial biogenesis, improving antioxidant defenses, or stabilizing mitochondrial dynamics (5).
